# A simple model of epileptic seizure propagation: Potassium diffusion versus axo-dendritic spread

**DOI:** 10.1371/journal.pone.0230787

**Published:** 2020-04-10

**Authors:** Anton V. Chizhov, Aleksei E. Sanin

**Affiliations:** 1 Computational Physics Laboratory, Ioffe Institute, Saint Petersburg, Russia; 2 Laboratory of Molecular Mechanisms of Neural Interactions, Sechenov Institute of Evolutionary Physiology and Biochemistry of the Russian Academy of Sciences, Saint Petersburg, Russia; 3 Blue Brain Project, École polytechnique fédérale de Lausanne (EPFL), Campus Biotech, Geneva, Switzerland; Georgia State University, UNITED STATES

## Abstract

The mechanisms of epileptic discharge generation and spread are not yet fully known. A recently proposed simple biophysical model of interictal and ictal discharges, Epileptor-2, reproduces well the main features of neuronal excitation and ionic dynamics during discharge generation. In order to distinguish between two hypothesized mechanisms of discharge propagation, we extend the model to the case of two-dimensional propagation along the cortical neural tissue. The first mechanism is based on extracellular potassium diffusion, and the second is the propagation of spikes and postsynaptic signals along axons and dendrites. Our simulations show that potassium diffusion is too slow to reproduce an experimentally observed speed of ictal wavefront propagation (tenths of mm/s). By contrast, the synaptic mechanism predicts well the speed and synchronization of the pre-ictal bursts before the ictal front and the afterdischarges in the ictal core. Though this fact diminishes the role of diffusion and electrodiffusion, the model nevertheless highlights the role of potassium extrusion during neuronal excitation, which provides a positive feedback that changes at the ictal wavefront the balance of excitation versus inhibition in favor of excitation. This finding may help to find a target for a treatment to prevent seizure propagation.

## 1 Introduction

Epilepsy is characterized by repeated seizures associated with abnormal intense electrical neural discharges. Despite ongoing research, the mechanisms of the generation and propagation of these discharges are not yet fully understood. Understanding these mechanisms is important for medical treatment development and helpful for mathematical modeling as an explanatory example of neuronal synchronization, which is a simpler regime of activity than normal functioning. On the other hand, in contrast to normal brain simulations, epileptic discharges involve the dynamics of ionic concentrations, thus requiring a more complex mathematical description.

The spread of activity through cortical circuits has been studied in experiments by means of electrical registrations and optical imaging [1–3], and high-density microelectrode arrays [4]. Experiments show slow propagation of an ictal wavefront and fast spread of discharges behind the front [3] [5]. The ictal wavefront progresses through the cortical area at a pace of < 1 mm/s, which is consistent with propagation speeds measured with electrodes and imaging in brain slice models [1, 2, 6–9] and *in vivo* (0.6 mm/s in [10] with two-photon microscope and 0.5 mm/s in [11] with widefield imaging in mouse neocortex). In the wake of the ictal wavefront, firing bursts are observed that are highly coherent across microelectrode recording sites within the same region, and correlated with ictal discharges recorded from adjacent macroelectrodes. The area demonstrating this hypersynchronized bursting is termed the ictal core [5].

The mechanism of the propagation is still an open question [3]. The main hypotheses consider the diffusion of potassium ions, the synaptic interactions, the electric field interactions, or the potassium electrodiffusion. As shown, the major role in excitability belongs to the extracellular potassium concentration. Recently, the spatial patterns of the extracellular potassium distribution have been registered by means of a nanoparticle-based technique [12]. The wavefront of potassium elevation from a seizure in the 4-AP (4-aminopyridyne, which strengthens synaptic connections) based model of cortical epilepsy spreads with a speed of about tenths of millimeters per second, which is similar to the typical speed of the ictal front. However, whether the potassium ion spread only accompanies the ictal front or explains the spread of the ictal front by diffusion or electrodiffusion is still a contested question. On one hand, observations of interictal discharges in slices show that correlated discharges exist even in the case of a lesion across a slice and are blocked after prevention of diffusion [13], thus pointing to non-synaptic, presumably potassium diffusion-based origin of the activity propagation or synchronization. On the other hand, the speed of potassium waves due to diffusion is limited [14, 15], and it is uncertain if the potassium diffusion-based hypothesis and simulations [16] are consistent with the experimental estimations. Moreover, the mentioned experiments [13] describe not the behaviour of the ictal front but only the bursts inside the domain of spontaneous activity generation. In the present paper, by means of simulations we show that the potassium diffusion may cause the propagation of the excitation that leads to the discharge generation but do not determine the speed of ictal wavefronts.

Synaptic communications seem to play a dominant role in ictal front propagation. That is, the seizure propagation respects the excitatory network of connectivity underlying normal processing [11], and brain slice data shows that ahead of the ictal wavefront there are very large feedforward excitatory and inhibitory synaptic conductances [1, 2, 4]. The ictal wavefront generates huge feedforward excitation, yet a rapid feedforward inhibition provides a powerful restraint. Seizures *in vivo* propagate as slowly as the ictal wavefronts in the *in vitro* zero-magnesium model. However, epileptiform events recorded in disinhibited slices propagate much faster, between 10 and 200 mm/s, presumably because of little or no effective feedforward inhibition to slow propagation [1]. The exact mechanism by which the restraint is overcome during recruitment of cortical territories to a seizure remains to be determined, but may involve activity-dependent mechanisms that either boost excitatory neurotransmission or compromise inhibitory neurotransmission, or both [4]. Here, we suppose that an activity-evoked elevation of the extracellular potassium level might be a key factor in the change of the balance of excitation versus inhibition at the ictal front.

Some mathematical models that consider spatial propagation suggest that the excitability of the tissue surrounding the seizure core may play a determining role in the seizure onset pattern [17]. Whereas the generation of interictal discharges is modeled in the conditions of impaired-but-fixed ionic concentrations [18], the dynamics of ictal discharges and the excitability of the cortical tissue is hypothesized to be governed by the ionic dynamics [19], [20], [21]. Generally, a computational approach to this issue requires a biophysical consideration of the neuronal population interactions in the conditions of changing ionic concentrations of sodium, potassium, chloride, and calcium ions inside and outside the neurons and glial cells. This problem is quite complex and computationally expensive. The most well-elaborated biophysical models consider either a single neuron [22], or a network without a spatial structure [23, 24], or a spatially structured but synaptically uncoupled neuro-glial network [19]. Taking into account the spatial propagation adds extra independent coordinates and thus essentially increases the complexity of models. For this reason, a consideration of spatial propagation requires a reduced but biophysically plausible model able to reproduce ictal discharges. Recently, we have proposed a spatially concentrated biophysical model of ictal and interictal discharges [25], called Epileptor-2 after the known abstract model Epileptor [26] that was further extended to large-scale simulations [27], [28]. Our model might also be extended to the spatially distributed case. In the present work, the Epileptor-2 model is extended by introducing the diffusion equation for the potassium concentration and the equation of spatial communications of neuronal populations via firing and postsynaptic propagation along axonal and dendritic trees [29].

## 2 Materials and methods

**Fig 1:**
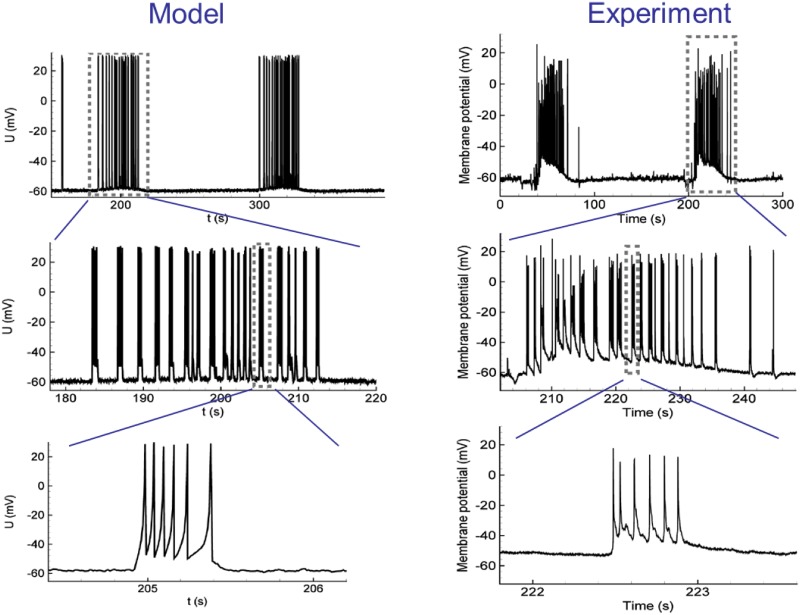
Single neuron activity during ictal discharges in simulation with the orignal Epileptor-2 model (left) and experiment (right). Two ictal discharges as bursts of clustered interictal-like short bursts are seen in the membrane voltage. In the experiment, the ictal discharges were recorded in a pyramidal neuron from a rat entorhinal cortex using an *in vitro* 4-AP model of epileptic activity. Modified from [21].

The previous implementation of Epileptor-2 [25] was restricted to the consideration of a spatially homogeneous activity. A primary advantage of that model is a biophysical interpretation of its governing variables that describe the pathological states of brain activity. For that purpose, the ionic dynamics equations used in earlier studies [23, 30, 31] are incorporated into a rate-based model for recurrently connected excitatory and inhibitory neuronal populations. The ionic dynamics description comprises equations for the concentrations of extracellular potassium and intracellular sodium. The firing rate of the inhibitory population is assumed to be proportional to that of the excitatory population, and thus, the inhibitory population is taken into account implicitly. The firing rate is described as a rectified sigmoid function of a membrane potential. The membrane potential is described by Kirchoff’s current conservation law, which is written for a one-compartment neuron. The expressions for the excitatory and inhibitory synaptic currents, the input-output function, the rate-based equations for the ionic dynamics, etc., are justified in [25]. The short-term synaptic depression is described according to the Tsodyks-Markram model. An adaptive quadratic integrate-and-fire model is used to reveal a spiking activity of a representative neuron. Behavior of such representative neuron is quite similar to that of a real neuron during generation of ictal discharges (Fig 1).

For the spatially inhomogeneous case, the model is supplied with the equations for the extracellular potassium ion diffusion and the spike and synaptic current propagation along neuronal axons and dendrites. As in the previous implementation, the full system of equations is split into three subsystems that describe (i) the ionic dynamics, (ii) the neuronal excitability, and (iii) a neuron-observer. To be explained below, the equations for the balance of the extracellular potassium and intracellular sodium concentrations, [*K*]_*o*_ and [*Na*]_*i*_, are as follows:

$$\begin{array}{ccc} \frac{\partial^{}{\left[K\right]}_{o}}{\partial t^{}} & = & D_{K}(\frac{\partial^{2}{\left[K\right]}_{o}}{\partial x^{2}}+\frac{\partial^{2}{\left[K\right]}_{o}}{\partial y^{2}})+\frac{{\left[K\right]}_{bath}-{\left[K\right]}_{o}}{\tau_{K}}-2\gamma \;I_{pump}\left(t\right)+\delta_{K}\theta \left(t\right), \end{array}$$ (1)

$$\begin{array}{ccc} \frac{d{\left[Na\right]}_{i}}{dt} & = & \frac{{\left[Na\right]}_{i}^{0}-{\left[Na\right]}_{i}}{\tau_{Na}}-3I_{pump}\left(t\right)+\delta_{Na}\theta \left(t\right). \end{array}$$ (2)

The equation for the membrane potential *V* is

$$\begin{array}{c} C\frac{dV}{dt}=-g_{L}V+u\left(t\right) \end{array}$$ (3)

The equation for the synaptic resource *x*^*D*^ is

$$\begin{array}{c} \frac{dx^{D}}{dt}=\frac{1-x^{D}}{\tau_{D}}-\delta_{x}\;x^{D}\varphi \left(t\right) \end{array}$$ (4)

The equation of spatial communications of neuronal populations via firing and postsynaptic propagation along axonal and dendritic trees is written for the presynaptic rate *φ*(*t*) governed by the somatic rate *ν*(*t*):

$$\begin{array}{c} \frac{\partial^{2}\varphi }{\partial x^{2}}+\frac{\partial^{2}\varphi }{\partial y^{2}}=\frac{\varphi -\nu }{\lambda^{2}} \end{array}$$ (5)

In the above equations, *θ*(*t*) represents the firing rate affecting potassium and sodium concentrations. It is different for each model of the mechanism of propagation (Models 1, 2, and 3; see the Results section). It is equal to either *ν*(*t*) or *φ*(*t*), where *ν*(*t*) is the firing rate on somas, and *φ*(*t*) is the firing rate on presynapses:

$$\begin{array}{c} \theta (t)=\nu (t)\;\text{(for}\;\text{Model}\;\text{1)}\;\;\;\;\text{or}\;\;\;\;\theta (t)=\varphi (t)\;\text{(for}\;\text{Models}\;\text{2}\;\text{and}\;\text{3)} \end{array}$$ (6)

The somatic firing rate *ν* is calculated with a sigmoidal input-output function, where [*x*]_+_ is equal to *x* for the positive argument and 0 otherwise:

$$\begin{array}{c} \nu \left(t\right)=\nu_{max}{\left[\frac{2}{1+\exp[-2\left(V\left(t\right)-V_{th}\right)/k_{v}]}-1\right]}_{+} \end{array}$$ (7)

The input current *u*(*t*) includes the potassium depolarizing current, the synaptic drive, and the noise *ξ*, respectively:

$$\begin{array}{c} u\left(t\right)=g_{K,leak}\left(V_{K}\left(t\right)-V_{K}^{0}\right)+G_{syn}\varphi \left(x^{D}\left(t\right)-c_{IE}\right)+\sigma \xi \left(t\right) \end{array}$$ (8)

The potassium reversal potential is obtained from the ion concentrations via the Nernst equation:

$$\begin{array}{c} V_{K}\left(t\right)=26.6mV\ln(\frac{{\left[K\right]}_{o}}{130mM}) \end{array}$$ (9)

The functional form of *Na*^+^/*K*^+^ pump current is taken from [31] (see also [32]) with modifications from [22]:

$$\begin{array}{c} I_{pump}\left(t\right)=\frac{\rho }{\left(1+\exp(3.5-{\left[K\right]}_{o}))(1+\exp((25-{\left[Na\right]}_{i})/3)\right)} \end{array}$$ (10)

Eq (1) includes the following terms: (i) the lateral diffusion term, (ii) the relaxation of [*K*]_*o*_ to the bath concentration due to diffusion and glial buffering, (iii) the potassium influx due to ATP-dependent Na-K pump and (iv) the potassium elevation proportional to the firing rate, which cumulatively approximates the effects of the potassium-chloride cotransporters, the voltage-gated channels, and glutamatergic synapses, whose activation is roughly proportional to the firing rate. Eq (2) includes (i) the [*Na*]_*i*_ relaxation term describing leakage and intracellular diffusion, (ii) the outflow due to the ATP-dependent Na-K pump, and (iii) the [*Na*]_*i*_ elevation during the firing activity by means of the voltage-dependent and glutamatergic channels. Eq (3) describes a mean membrane depolarization due to the input *u*(*t*) using a single-compartment leaky neuron model. Not taking into account a spike generation, the voltage *V*(*t*) reflects a nominal, extreme level of membrane polarization. Together with Eq (8), it is a stochastic ordinary differential equation for the Ornstein-Uhlenbeck process. Eq (8) takes into account (i) the depolarizing effect of the potassium concentration increase relative to its initial concentration, (ii) the total synaptic current, and (iii) the noise. The current reflects the indirect effects of the ionic concentrations on membrane polarization through the voltage-gated and leak channels. The first term is a linearization of the dependence of all potassium channels (mainly, the leak) on the reversal potential deflection $V_{K}\left(t\right)-V_{K}^{0}$. The synaptic term consists of two parts, positive *G*_*syn*_*φ*(*t*)*x*^*D*^(*t*) and negative −*c*_*IE*_ *G*_*syn*_*φ*(*t*). The positive component is an excitatory, short-term depressive current proportional to the presynaptic firing, thus implying an instantaneous synaptic kinetics. The short-term plasticity in the negative, inhibitory term is neglected; the coefficient *c*_*IE*_ sets the balance of inhibition versus excitation. Eq (4) describes the short-term synaptic depression according to the Tsodyks-Markram model written in the rate-dependent form [33].

This new spatially extended version of the model includes the diffusion term *D*_*K*_(∂^2^[*K*]_*o*_/∂*x*^2^ + ∂^2^[*K*]_*o*_/∂*y*^2^) in the equation for [*K*]_*o*_, Eq (1), and the equation for the presynaptic firing rate, Eq (5). Eq (5) implies that the spatial communications of neuronal populations is provided by two factors: (i) the spread of action potentials through axons and (ii) the spatial integration of postsynaptic currents at the dendritic branches. Taking into account only local isotropic connections and implying that the dendritic propagation affects the presynaptic firing rate attributed to somatic location, the presynaptic firing rate *φ* is obtained as a convolution of the somatic firing rate *ν* with the exponentially decaying kernel exp(−*r*/λ), where *r* is the distance between pre- and postsynaptic neurons and λ is the characteristic length of the connectivity profile. Alternatively, Eq (5) can be derived from the mean field equations proposed in [29, 34], assuming that the axonal delay is negligible. In our consideration, the axonal delay is much smaller than the membrane time constant which is the smallest time scale in the Epileptor-2 model, and thus it is to be neglected, which leads to Eq (5). We used the Neumann boundary conditions for Eqs (1) and (5), i.e. the fluxes of [*K*]_*o*_ and *φ* were zeroed at all the boundaries.

The representative neuron is modeled with an adaptive quadratic integrate-and-fire neuron [35]. The equations for its membrane potential *U* and adaptation current *w* are as follows:

$$\begin{array}{c} C_{U}\frac{dU}{dt}=g_{U}\left(U-U_{1}\right)\left(U-U_{2}\right)-w+u+I_{a} \end{array}$$ (11)

$$\begin{array}{c} \tau_{w}\frac{dw}{dt}=-w \end{array}$$ (12)

$$\begin{array}{c} \text{if}\;U>V^{T}\;\text{then}\;U=V_{reset},w=w+\delta w \end{array}$$ (13)

Most of the parameters were set as in the previous version of Epileptor-2 [25]. They are given in Table 1. A few values were modified (given in bold font in Table 1).

**Table 1:** Parameters of the models. Basic parameter values are from [25], except the modified values that are given in bold.

Parameter	Value with units	Description
*τ*_*K*_	100s	Potassium time constant
*τ*_*Na*_	20s	Sodium time constant
*C*/*g*_*L*_ or *τ*_*m*_	10ms	Membrane time constant
*τ*_*D*_	2s	Synaptic depression time constant
*D*_*K*_	**4 ⋅ 10^−6^**cm^2^/s	Potassium diffusion coefficient
*δ*_*K*_	**0.04**mM	Potassium concentration increment at spike
*δ*_*Na*_	0.03mM	Sodium concentration increment at spike
*δ*_*x*_	0.01	Synaptic concentration increment at spike
*ξ*	N(0,1)	Gaussian white noise
*σ*/*g*_*L*_	25mV	Noise amplitude
*ρ*	0.2mM/s	Maximum pump flux
*γ*	**20**	Volume ratio
*G*_*syn*_/*g*_*L*_	5mV⋅s	Postsynaptic charge
*c*_*IE*_	0.5	Inhibitory vs. excitatory conductance ratio
*g*_*K*,*leak*_/*g*_*L*_	**1.0**	Potassium leak conductance
${\left[K\right]}_{o}^{0}$	3mM	Initial extracellular potassium concentration
[*K*]_*bath*_	**7**mM	Extracellular potassium concentration
${\left[Na\right]}_{i}^{0}$	10mM	Initial intracellular sodium concentration
*ν*_*max*_	100Hz	Soma maximal firing rate
λ	**0.385**mm	Spatial scale of cortical fiber connectivity
*V*_*th*_	25mV	Threshold potential
*k*_*ν*_	20mV	Gain
*g*_*U*_	1.5nS/mV	Representative neuron conductivity
*C*_*U*_	**1050**pF	Representative neuron capacity
*V*^*T*^	25mV	Representative neuron threshold potential
*V*_*reset*_	-40mV	Representative neuron reset potential
*U*_1_	-60mV	Representative neuron reversal potential 1
*U*_2_	-40mV	Representative neuron reversal potential 2
*U*^0^	-70mV	Representative neuron initial potential
*I*_*a*_	116pA	Representative neuron tonic current
*τw*	200ms	Adaptation current time constant
*δw*	100pA	Adaptation current increment ap spike

### Justification of main assumptions

The model describes only intracellular concentration of sodium and extracellular of potassium. First, the concentrations of intracellular potassium and extracellular chloride and sodium ions are assumed to be constant, because their effect on the network is much smaller than that for their counterparts. As in [21], we estimate the sensitivity of the network to changes of the concentrations. We evaluate the sensitivity with the absolute values of the derivatives of the reversal potentials on the concentrations, i.e. |∂*V*_*ion*_/∂[*ion*]| = 26.6mV/[ion]. With [*Cl*]_*o*_ = 130mM, [*K*]_*i*_ = 130mM, [*Na*]_*o*_ = 130mM, [*Cl*]_*i*_ = 10mM, and [*K*]_*o*_ = [*K*]_*bath*_ and ${\left[Na\right]}_{i}={\left[Na\right]}_{i}^{0}$, we obtain |∂*V*_*K*_/∂[*K*]_*i*_| = 0.2mV/mM, |∂*V*_*Cl*_/∂[*Cl*]_*o*_| = 0.2mV/mM and |∂*V*_*Na*_/∂[*Na*]_*o*_| = 0.2mV/mM, which are about one order of magnitude smaller than |∂*V*_*K*_/∂[*K*]_*o*_| = 3.8mV/mM, |∂*V*_*Cl*_/∂[*Cl*]_*i*_| = 2.7mV/mM and |∂*V*_*Na*_/∂[*Na*]_*i*_| = 2.7mV/mM, correspondingly. These estimates justify the assumption of constant [*Cl*]_*o*_, [*K*]_*i*_, and [*Na*]_*o*_. Second, our study associates the intracellular chloride concentration changes with the extracellular potassium, thus keeping only the extracellular potassium and the intracellular sodium concentrations. The assumption is supported by experimental registrations of [*Cl*]_*i*_ and [*K*]_*o*_ during an ictal event, which show similar behaviour of these variables [36]. This issue and other assumptions are discussed in more details in [25].

We do not introduce into the models an electrodiffusion of potassium ions. In fact, the electric field produced by membrane currents on excited neurons at the front of the ictal discharge may accelerate the potassium ions in the extracellular space. This effect might be taken into account by the extra term *u*_*K*_*V*′ ∂[*K*]_*o*_/∂*x* in the equation for the extracellular potassium concentration, Eq (1), where *u*_*K*_ is the ion mobility and *V*′ is the voltage gradient [37]. Not considering the term with the second order derivatives, the equation would be a transfer equation of the form ∂[*K*]_*o*_/∂*t* − *u*_*K*_*V*′ ∂[*K*]_*o*_/∂*x* = *r*.*h*.*s*., where “r.h.s.” is the right hand side of Eq (1); the factor *u*_*K*_ *V*′ determines the speed of propagation of [*K*]_*o*_ perturbations. Taking the value for *u*_*K*_ from [37] to be equal to 5 ⋅ 10^−4^ *cm*^2^/(*s* ⋅ *V*) and estimating the voltage gradient to be 2*mV* per 100*μm*, based on local field recordings [4], we obtain the speed to be of an order of a few micrometers per second, which is negligibly small in comparison to the seizure propagation speed. This result means that the electrodiffusion is too weak to contribute significantly to the potassium spread and can be omitted.

### Numerical scheme and computer model

**Fig 2:**
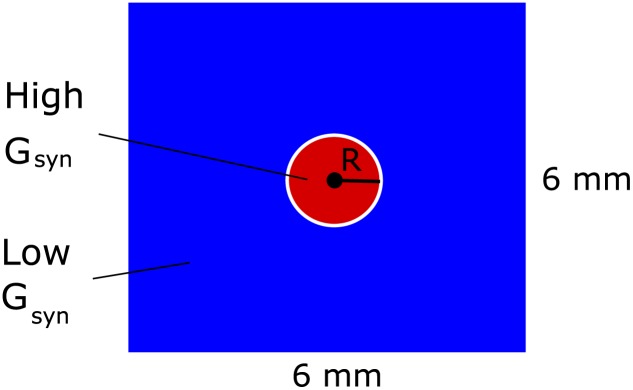
Computational domain includes a 6mm by 6mm piece of the cortical tissue. The central circular domain with *R* = 0.3 mm (red) is a center of excitation due to higher synaptic conductance *G*_*syn*_ than outside the circle (blue).

The simulations were performed in the Python 3.6 environment. The spatial derivatives in Eqs (1) and (5) have been approximated with the central difference scheme on a regular mesh with Neumann boundary conditions. The Euler-Maruyama explicit numerical scheme was applied for the integration in time of the stochastic ordinary differential equations and Eq (1). The linear algebraic system corresponding to the discretized Eq (5) was solved by the Jacobi method. The typical value of a time step was 1 ms. The computational domain was represented as a square with a side 6 mm and the circular center of excitation with *R* = 0.3 mm (Fig 2). The domain was discretized with a grid of 80x80 cells. The results were not significantly dependent on the numerical parameters and realizations of noise. The numerical realization of the model is available from the website gin.g-node.org/asanin/epilepsy-potassium-calculation.

## 3 Results

We have hypothesized two different mechanisms of the spatial propagation of epileptic activity. The first mechanism is based on the diffusion of potassium in the extracellular medium. The second is determined by the spread of spikes and synaptic currents through axons and dendrites isotropically distributed within the cortex. In order to distinguish between the two mechanisms, we have considered two different models of spatial propagation. Both models generalize Epileptor-2, the recently proposed spatially homogeneous model of epileptic activity [25]. The first model supplies Epileptor-2 with the diffusion term in the equation for the extracellular potassium concentration, Eq (1). Below we refer to this model as “the diffusion model,” or Model 1. The second model adds to the system the equation of spiking activity spread, Eq (5), which connects presynaptic to somatic firing rates by assuming an exponentially decaying profile of connectivity with some characteristic length of the connections. Below we refer to this model as “the synaptic model,” or Model 2.

Our simulations are aimed to reproduce the spatial-temporal patterns of activity of the cortical neural tissue during the generation of epileptic ictal discharges after local application of the proepileptic agent 4-AP [12]. In our models, we consider a square-shaped domain of nervous tissue with a small circular central zone being a source of epileptic discharges. In the central zone (Fig 2), the excitability *G*_*syn*_ is elevated by setting *G*_*syn*_/*g*_*L*_ = 5mV⋅s in contrast to *G*_*syn*_/*g*_*L*_ = 1mV⋅s at the periphery. Both models are stochastic due to the introduced spatially homogeneous noise in Eq (8).

### 3.1 Temporal aspects of activity in the center of epileptic discharge generation

**Fig 3:**
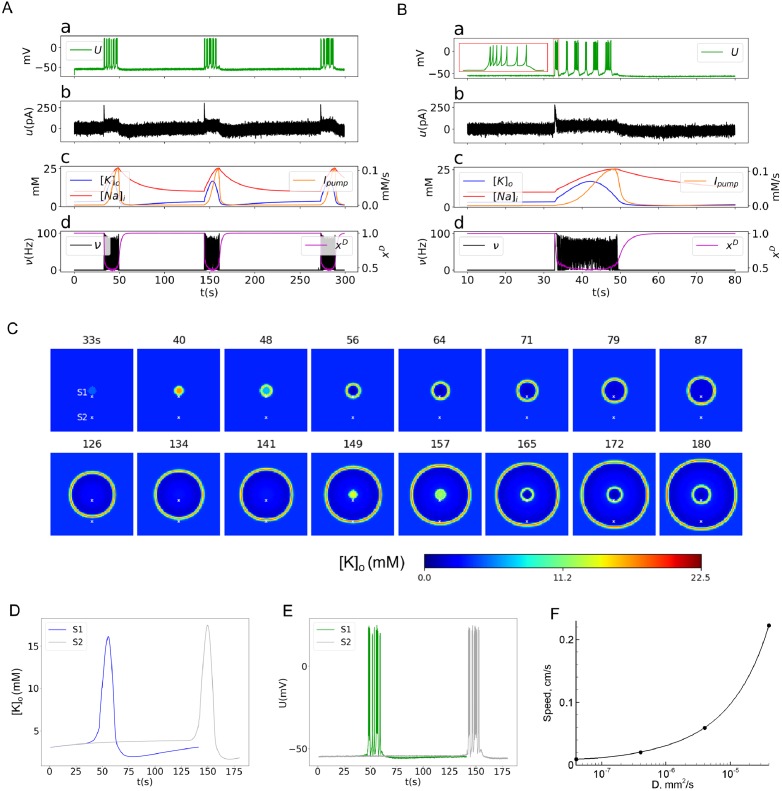
Diffusion mechanism (Model 1). A: Repeated IDs recorded in the center of the cortical domain (Fig 2). B: An ictal discharge recorded in the center of the cortical domain. a: The representative neuron membrane depolarization *U*. The inset is a magnification of the first burst. b: The total input current *u*. c: The ionic concentrations [*K*]_*o*_ and [*Na*]_*i*_, and the *Na*^+^/*K*^+^ pump current *I*_*pump*_. d: The somatic firing rate *ν* and the synaptic resource *x*^*D*^. C: Potassium concentration spatial-temporal patterns during generation of two IDs. Two sites of “recordings” are marked as “S1” and “S2”, remote at a distance of 2mm. D: A comparative plot of [*K*]_*o*_ at two points S1 and S2. E: A comparative plot of *U* at the sites S1 and S2. F: The dependence of the front wave speed on the diffusion coefficient (obtained with a narrow computational domain).

The two spatially distributed models show patterns of activity in the center of epileptic discharge generation similar to those in the original spatially homogeneous model Epileptor-2 [25]. Ictal (ID) and interictal discharges (IID) are reproduced. In the case of IDs, these quasi-periodic spontaneous events occur with an interburst interval of an order of minutes (Fig 3A). Each ID is characterized by a high-rate spiking activity, lasts about a few tens of seconds and consists of short bursts that resemble IIDs (Figs 3Ba and 4Ba); i.e. ID is a cluster of IID-like bursts. The membrane potential of the representative neuron (Figs 3Aa, 3Ba, 4Aa and 4Ba) and the concentrations of potassium and sodium ions (Fig 3Ac and 3Bc) reflect the spontaneous occurrence of the discharges. [*K*]_*o*_ dynamics determines the onset and the duration of an ID. The ID begins as soon as the slowly increasing [*K*]_*o*_ reaches a certain threshold level (Figs 3Bc and 4Bc). Then, [*K*]_*o*_ increases rapidly, because of intensive potassium extrusion through potassium voltage-gated and glutamatergic channels that are active during the ID; the activity of these channels is reflected in the firing rate (Figs 3Bd and 4Bd). [*K*]_*o*_ grows until it is balanced by the Na-K pump. The peak of [*K*]_*o*_ takes place at the middle of the ID. After that, [*K*]_*o*_ begins to decrease, finally returning to its baseline and even lower. The phase of the ID in which [*K*]_*o*_ concentration approaches the baseline determines the termination of the ID. The Na-K pump is activated by the elevated intracellular sodium concentration. The sodium concentration increases because of high spiking and glutamatergic synaptic activity during IDs [21]. When a certain high level of the intracellular sodium concentration is reached, the potassium-sodium pump activates (Figs 3Bc and 4Bc). The *Na*^+^/*K*^+^ pump peaks at the end of the ID. Its activity remains high until the baseline potassium concentration is restored. Then, the burst terminates, and the sodium concentration slowly decays to the original concentration before the next ID (Figs 3Ac and 4Ac).

**Fig 4:**
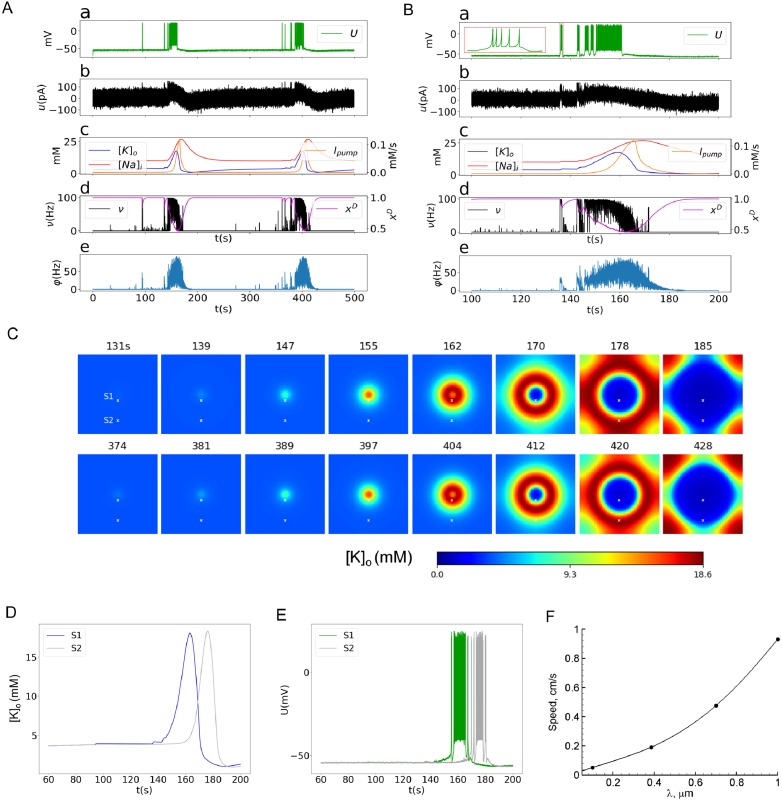
Synaptic mechanism (Model 2). A: Repeated IDs recorded in the center of the cortical domain(Fig 2). B: A single ictal discharge recorded in the center of the cortical domain. a: The representative neuron membrane depolarization *U*. The inset is a magnification of the first burst. b: The total input current *u*. c: The ionic concentrations [*K*]_*o*_ and [*Na*]_*i*_, and the *Na*^+^/*K*^+^ pump current *I*_*pump*_. d: The somatic firing rate *ν* and the synaptic resource *x*^*D*^. e: The presynaptic firing rate *φ*. C: Potassium concentration spatial-temporal patterns during generation of two IDs. D: A comparative plot of [*K*]_*o*_ at two different points. E: A comparative plot of *U* at two different points. F: The dependence of the front wave speed on the connection length (obtained with a narrow computational domain).

From a mathematical point of view, the dynamics of IDs is determined by the extracellular potassium and intracellular sodium ionic concentrations which dynamics is close to slow deterministic oscillations, as shown in [25]. It is governed by a slow subsystem of the full system of equations, based on Eqs (2) and (1) without the diffusion term, both dependent on a time averaged firing rate that is a function of [*K*]_*o*_. On the contrary, the IID-like events that constitute IDs are governed by a fast subsystem, based on Eqs (3) and (4). These events represent spontaneous large-amplitude oscillations (Figs 3Ba and 4Ba).

### 3.2 Spatial aspects in Model 1: Diffusion

In the consideration of the extracellular potassium diffusion-based mechanism of activity spread, simulations were conducted with the diffusion equation Eqs (1) and (6) set to be *θ* = *ν*, thus omitting Eq (5). The diffusion coefficient was taken from [14].

The model’s behavior at the central point is qualitatively similar to the spatially homogeneous case [25] and differs only quantitatively. The quasi-periodic spontaneous IDs occur at an interval of about 110-130s (Fig 3A). Each ID is characterized by a high rate of activity lasting about 17s (Fig 3B) and consisting of short bursts resembling IIDs (Fig 3Ba). The potassium threshold for ID initiation is about 4mM. Initiated at the center, the ID forms a radial wave, which spreads across the entire cortical domain, as seen from patterns of the extracellular potassium concentration (Fig 3C). [*K*]_*o*_ rapidly increases at the front of the wave and more gradually decreases after the activation of the Na-K pump due to increasing sodium concentration, thus constituting the rear phase of the wave. [*K*]_*o*_ finally returns to its baseline and even lower. After reaching its minimum, the potassium concentration slowly increases toward the threshold of another ID initiation.

The wave profile remains approximately the same during its propagation, as seen from a comparison of [*K*]_*o*_ evolution at two sites (Fig 3D), located in the center and the periphery and marked in Fig 3C. The moving pulse of [*K*]_*o*_ corresponds to a single ID, formed as a cluster of IID-like discharges seen in the voltage plot in Fig 3E. The ID duration is approximately the same at the two sites. The amplitude of the [*K*]_*o*_ pulses varies within 10% (Fig 3D). At the same time, the voltage patterns of IIDs are different, reflecting the spontaneous bursting character of the activity.

The velocity of the first potassium wave is about 0.035 mm/s. The second wave initiates at about 149s (Fig 3D). Its velocity is roughly the same, about 0.03 mm/s. The acceleration is due to the higher level of [*K*]_*o*_ in front of the second wave, as seen from the comparison of the shapes of the first and second IDs detected at the same site S1 (Fig 3D). Still, the velocities obtained in physiological range of the diffusion coefficient values (Fig 3F) are much smaller than the experimental measures.

### 3.3 Spatial aspects in Model 2: Axo-dendritic spread

In the consideration of the synaptic mechanism of activity spread, simulations were conducted with the equation for the axo-dendritic propagation of spiking activity, Eqs (5) and (6) set to be *θ* = *φ*, thus without the diffusion term in Eq (1).

The model’s behavior at the central point is also qualitatively similar to the spatially homogeneous case [25] and the previous scenario (Section 3.2), with only quantitative differences. The quasi-periodic spontaneous IDs occur at an interval of about 220s (Fig 4A). Each ID is characterized by a high rate of activity lasting about 40s (Fig 4B) and consisting of short bursts resembling IIDs (Fig 4Ba). The potassium threshold for ID initiation is about 4mM. The spatial propagation is qualitatively similar to that in simulations with Model 1. Initiated at the center, the ID forms a radial wave, which spreads across the entire cortical domain (Fig 4C). [*K*]_*o*_ rapidly increases at the front of the wave and more gradually decreases after the activation of the Na-K pump due to increasing sodium concentration (Fig 4Bc), thus constituting the rear phase of the wave. [*K*]_*o*_ finally returns to its baseline and even lower (shots at 170s and later in Fig 4C). After reaching its minimum, the potassium concentration slowly increases toward the threshold of another ID initiation after about 381s (Fig 4A and 4C).

The velocity of the first potassium wave is about 0.11 mm/s. The second moves with roughly the same velocity (Fig 4C). The speed is roughly proportional to the spatial scale of spatial connections λ (Fig 4F), being also dependent on the geometrical scale of the domain of excitation. The speed value is quite consistent with the range of experimental measures mentioned in the Introduction section. For instance, both the speed and the spatial scale are comparable with the speed and the size of neuronal clusters active during the discharges registered in [9].

The wave profile remains approximately the same during the propagation, as seen from a comparison of [*K*]_*o*_ evolution at two sites (Fig 4D), located in the center and the periphery, and marked in Fig 4C. The moving pulse of [*K*]_*o*_, shown in Fig 4D, corresponds to a single ID. At different points of time, the same ID forms different clusters of IID-like discharges seen in the voltage plot in Fig 4E. These clusters of spike bursts are different at the two sites, because of the spontaneous generation of IID-like events.

**Fig 5:**
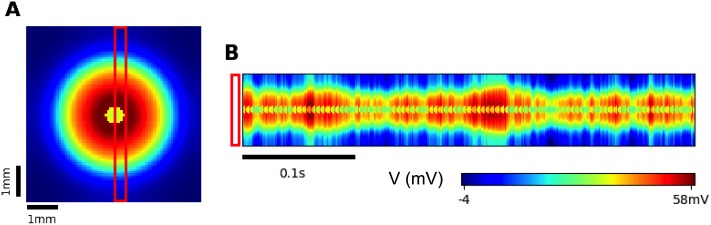
Synaptic mechanism (Model 2). A. The membrane potential *V* at the 163rd second of the simulation. The red bar marks the region of interest (ROI) for spatial-temporal analysis. B. Propagation pattern of the ROI within the interval of 400 ms since the time moment at 163s. The stripes-bursts constituting the pattern are rather vertical, which means that the speed of the bursts is much larger (or infinite) in comparison to that of the ictal wave.

To clarify the spatial character of IID-like event generation and propagation, we have outlined a region of interest (ROI) in the form of a vertical strip aligned across the center of the simulation area (Fig 5A). The diagram of the activity in ROI versus the vertical coordinate *y* and time *t* (Fig 5B) demonstrates the effect of synchronization of the voltage bursts between the center of the ictal discharge generation zone and its periphery. During the time period of 0.4s, which is short in comparison with the time scales of the potassium wave propagation during an ID (Fig 4C), the voltage profile across *y* frequently fluctuates (Fig 5B). The fluctuations are seen as thin vertical stripes on the *y*−*t* diagram. Each such fluctuation is a burst that constitutes an ID. The outer envelope of the fluctuations, being averaged in time, corresponds to the front of the potassium wave. The fact that the bursts-stripes are vertical means that the activity within each stripe occurs simultaneously within some range of *y*. Alternatively, an event with a finite speed would have an inclined front. Thus the propagation of the voltage bursts is almost instantaneous in comparison with that of the potassium wave (Fig 4C).

**Fig 6:**
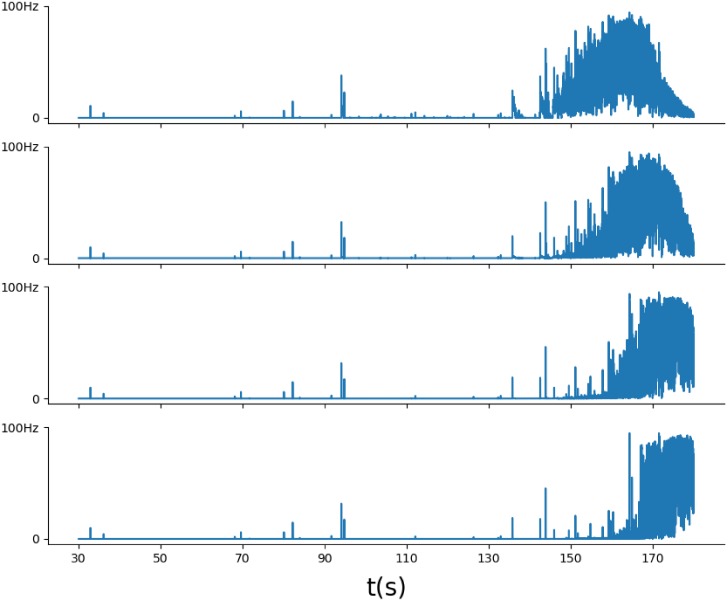
Preictal events at different points are highly correlated, whereas the onsets of ID are delayed. The firing rate *φ* is plotted at different points with the distance 1 mm between them.

This effect of essentially different speeds of the waves of slow variables and fast (the potassium concentration and the membrane potential, correspondingly) is quite consistent with the experimental observations from [38] (see Fig 6 there). In their experiment, the wave of an intrinsic optical signal that reflected the cerebral blood volume was much slower than the wave of a voltage-sensitive dye signal. Suggesting that the cerebral blood volume follows the extracellular potassium concentration, our simulation explains that the slow wave originates as an envelope of fast voltage bursts and travels with the speed of IDs. The speed of the slow waves in our simulation (0.15 mm/s) was also comparable to that in their experiment (0.45 mm/s).

Because of slow propagation, the delays between the onsets of IDs at the points remote on scale of a millimeter are as large as a few seconds (Figs 4D and 6). By contrast, the afterdischarges and preictal discharges are much more precisely correlated. Small firing rate bursts precede an ID (Fig 6). They are either independent IIDs (for example, see the bursts at about t = 95s) or those that correspond to early bursts belonging to an ID that begins at some distance. In the latter case, the events simply reflect the excitation happening within the core of an ID. In both cases, the events do not recruit neurons into active spike generation that constitutes an ID. The real recruitment, characterized by an intense, sharp increase in the firing rate, occurs later.

Model 2 shows essentially larger waves than Model 1, as seen from a comparison of the domains with high [*K*]_*o*_ in Fig 4C to those in Fig 3C. This difference is because of the higher speed and longer duration of IDs in Model 2. The large waves are more consistent with experiments. Also, the speed of the wave in Model 1 is too slow in comparison to measurements [1, 39, 40], which estimate a range of about 0.2-10 mm/s. These observations favor Model 2. The potassium diffusion mechanism is too slow to explain the ictal discharge propagation. In contrast, the propagation of spikes and synaptic currents by the axons and dendrites is a sufficient mechanism to explain the ictal discharge propagation.

As experiments have shown, the disinhibition dramatically increases the ictal wavefront speed up to 10 ÷ 200 mm/s [4, 41]. In our model, the contribution of the inhibition is determined by the factor *c*_*IE*_. In simulations, the decrease of this factor accelerates the propagation up to two orders of magnitude. (Variations of *c*_*IE*_ in the range 0.25 ÷ 0.8 preserve the wave-like solution and change the speed in 0.3 ÷ 20 times, as obtained in simulations with a narrow computational domain.) Therefore, the model is consistent with experiments in this aspect.

The crucial role of the depolarization provided by the elevation of [*K*]_*o*_ in the wavefront propagation is confirmed by the increase of the speed with the increase of *g*_*K*,*leak*_. The potassium-mediated positive feedback depends on the sensitivity of the membrane polarization to [*K*]_*o*_, evaluated by *g*_*K*,*leak*_. Hence the stronger the feedback, the faster the ictal wavefront. This model prediction has to be verified in further experiments.

**Fig 7:**
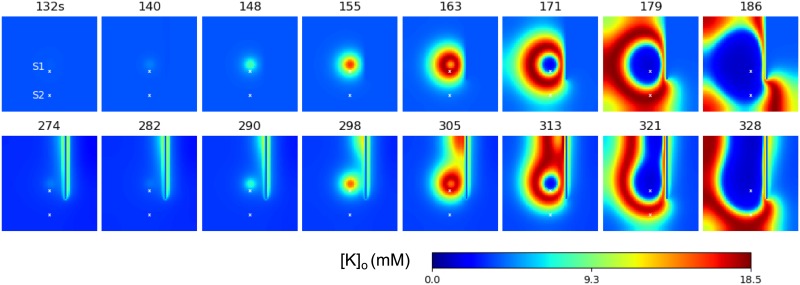
Model 2: Domain with a lesion. Potassium concentration spatial-temporal patterns during generation of two IDs.

If the propagation depends on the synaptic connections, then damage to the connections is able to prevent the activity spread. We demonstrate this with a simulation of activity spread in a domain with a straight lesion set with the condition *φ* = 0 on a line segment (Fig 7). The activity cannot propagate across the lesion. Instead, it passes around the lesion and spreads through the entire area behind.

### 3.4 Model 3: Combination of the two mechanisms of activity spread

Our finding that the axo-dendritic connections provide faster propagation of epileptic discharges than potassium diffusion does prompt us to check whether the same fast propagation takes place in the situation where the both mechanisms are functioning together. To this end, we have considered a model that incorporates the entire system of Eqs (1)–(13). As suggested, simulations with the combined model have shown results quite similar to those from Model 2, which were described in the subsection 3.3. Therefore, taking the potassium diffusion into account in Model 2 does not change the solution, thus proving that potassium diffusion does not affect the spread of IDs. Axo-dendritic spread prevails over potassium diffusion.

**Fig 8:**
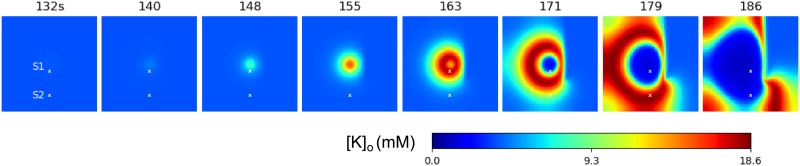
Model 3: Domain with a partial lesion. Potassium concentration spatial-temporal patterns during generation of a single ID.

In the case of the propagation in the domain with a lesion, as in the case shown in Fig 7 for the simulation with Model 2, the diffusion is potentially able to cross through the lesion, in contrast with the spiking activity which is stopped at the damage of the connections. However, within the time period of a single ID spread, the effects of the potassium diffusion are negligibly small, which is seen from a comparison of Figs 7 and 8 (compare the narrow regions of increased potassium near the lesion in two figures). Consequently, the potassium diffusion does not affect the spread of IDs.

**Fig 9:**
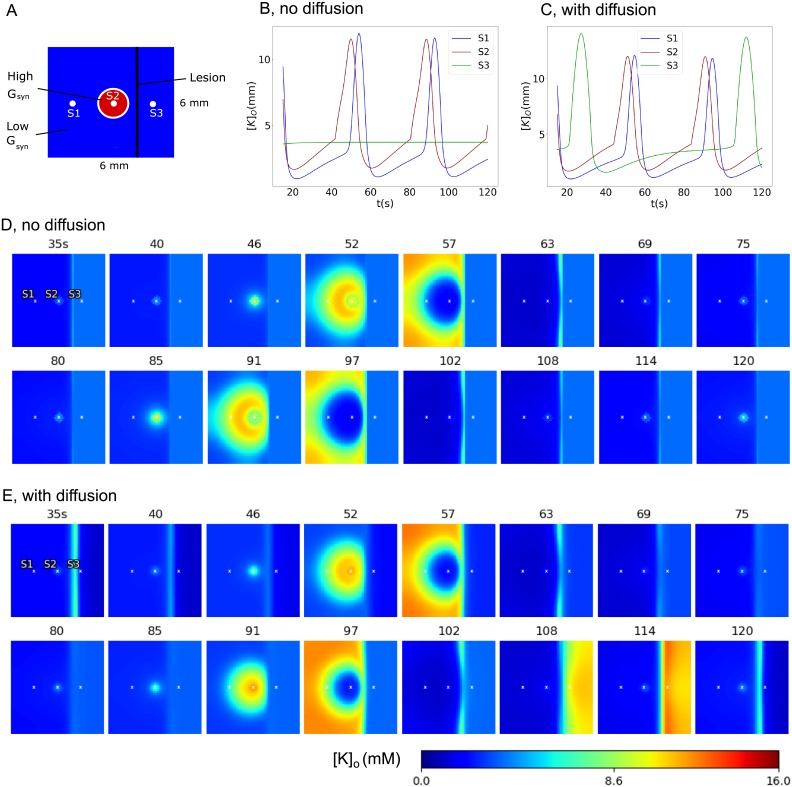
Model 3: Domain with a complete lesion. A. The schematic of the computational domain. B and C. The evolution of the extracellular potassium concentration at the three sites of the domain, S1, S2 and S3, in simulations with (C) and without (B) the diffusion. D and E. The spatial-temporal patterns of the extracellular potassium concentration during generation of two discharges in the left domain. The discharge in the right domain originates due to the potassium diffusion. Specific parameters were: *τ*_*K*_ = 10s, *g*_*K*,*leak*_/*g*_*L*_ = 3, *K*_*bath*_ = 4mM and *G*_*syn*_/*g*_*L*_ = 1mV⋅s in the periphery, *K*_*bath*_ = 7mM and *G*_*syn*_/*g*_*L*_ = 5mV⋅s in the central zone, a spatially nonhomogeneous noise, *D*_*K*_ = 2 ⋅ 10^−5^cm^2/^s.

The observed minor role of the potassium diffusion seems to be contradictory to the experiment performed by Lian et al. [13]. With a complete lesion of a slice by a blade they have detected epileptiform discharges in the two halves of the slice. The discharges were time-correlated, if the potassium diffusion was not impaired. The discharges were interictal-like ones, weaker and more frequent than ictal discharges. To reproduce the effect, we have set a complete lesion of synaptic connections on a line crossing the computational domain (Fig 9A). With the basic set of parameters, the diffusion could not affect the discharge propagation across the lesion. We have modified some parameters of sensitivity to potassium, excluded any possible spatial correlations due to noise by supplying a spatially non-homogeneous noise, and increased the potassium diffusion coefficient to its characteristic value in pure electrolytes. As a result, we observed discharges in the entire domain (Fig 9C and 9E), which were weaker and more frequent than those in the previous simulations (compare Fig 9C with Figs 3D and 4D). They originate in the central, more excitable region and spread rapidly within the left sub-domain. The elevated potassium diffuses through the lesion and evokes discharges in the sub-domain behind the lesion. With the diffusion blocked, the right sub-domain behind the lesion keeps silent (Fig 9B and 9D). Therefore, the diffusion of the potassium ions is able to excite nearby regions, but with a significant delay, and moreover this effect is observed in a narrow range of simulation parameters. Summarizing, the potassium diffusion is able to transfer excitation but do not determine the speed of ictal wavefronts.

## Discussion

With the help of a simple but biophysically meaningful model, we have considered two different hypotheses of ictal discharge propagation. The first one, Model 1, is based on the extracellular potassium diffusion. The second hypothesis, Model 2, is based on spatial propagation due to synaptic interactions. Both models reproduced ictal discharges as traveling waves that are similar to experimental ictal waves by many characteristics including the frequency, the duration, the composition of interictal-like bursts, the spontaneous character of bursts, the ionic dynamics and the dynamics of spiking. The main difference between the models is the speed of the waves. The diffusion model led to inconsistently thin and overly slow ictal waves, if the diffusion coefficient is constrained by its experimental estimations. Only an artificially increased diffusion coefficient may give comparable speeds of waves, as in [16], where the coefficient was 5 orders of magnitude higher than the real values (1 cm^2/^s). These observations are not in favor of the first hypothesis. On the contrary, the synaptic mechanism of propagation resulted in simulated ictal waves with a speed similar to experimental measurements in slices [1, 2, 6] as well as *in vivo* in animal models [10, 11] and in human patients [4, 5]. Further, we have shown that an addition of the diffusion mechanism to the synaptic one (Model 3) does not change the solution; i.e., the diffusion is too slow and cannot significantly contribute to the propagation. This comparison justifies the hypothesis that the synaptic transmission may fully determine the speed of the ictal wavefront.

Role of feedforward inhibition in seizure propagation

Current experimental studies of seizure propagation reveal the role of feedforward inhibition [1, 2, 4, 5, 9, 10]; however, a certain mechanism is still debatable [5]. During recruitment of new territories to an existing ictal event, the driving force is provided by the glutamatergic output of the seizing, “core” territories. Interneurons contribute to the feedforward inhibitory response. The ictal wavefront phenomenon coincides with a shift from predominately inhibitory to predominately excitatory currents. Ahead of this event, strong inhibitory currents effectively block pyramidal firing [1]. This “inhibitory veto” operates in the penumbra, limits the ictal wavefront’s advance, and thus determines the speed of the wavefront. This mechanism is consistent with the behavior of our Models 2 and 3, but there is one detail that is different in our explanation. It was hypothesized that a collapse of inhibition takes place at the ictal wavefront, which is, however, difficult to reveal directly from experiments [1]. Our Model 2 reveals that it is not necessary to assume any additive factor such as the collapse of inhibition in order to explain the changes at the ictal wavefront. At the same time, the model supported high sensitivity of the speed of the wavefront to the intensity of inhibition, such that disinhibition is able to significantly speed up the front, as in [41].

Role of potassium-mediated positive feedback in seizure propagation

Instead of feedforward inhibition, an increase of potassium concentration leads to the change in excitation-versus-inhibition balance. The potassium increases because of its outflux through voltage-gated and glutamatergic channels opened in the excited state at the ictal wavefront. This localized elevation of potassium concentration depolarizes the neuronal membranes, thus providing a positive feedback of excitation. This extra excitation changes the balance and forces the glutamatergic cells to fire and contribute to the common excitation underlying the ictal discharge. This scenario is consistent with the observations of highly correlated and fast onsets of electric activity and potassium increase [7].

Speed of preictal bursts and afterdischarges

The correlation of the onset moments of an ictal discharge at distal points is determined by the speed of the discharge. It is slow, and the delay between the signals at the points remote at a distance of about one millimeter is typically as large as a few seconds [4, 5] (for instance, see Fig. 4a in [4]). By contrast, the afterdischarges and preictal discharges are much more precisely correlated. In electrophysiological recordings by multiple electrodes [4], there was a marked discrepancy between the apparent onset time of the ictal rhythm and the actual time when neurons were recruited. The earliest low-frequency ictal rhythms propagated rapidly across all electrodes, however, there was only an irregular and relatively low level of unit activity, and the real recruitment, characterized by an intense sharp increase in unit activity, occurred only later [4]. Similar weak synchronous bursts were observed before the onset of an ictal discharge in Model 2, which were simply reflections of the bursts that are generated inside the ictal core and compose the ictal discharge. Also, the afterdischarges are well correlated if observed as local field potentials [38] or intracellular signals [42]. They are also synchronized inside the ictal core in Model 2. Therefore, Model 2 is consistent with the description of the spreading seizure in terms of the ictal core and the penumbra [4, 5].

A lesion prevents the ID propagation

Our simulation of a spread in a domain with a lesion has shown that an ID cannot directly propagate across lesions, but instead, it passes around them. This finding seems to be contradictory to some experiments in slices [13], where epileptiform discharges were found to be synchronized on both sides of a complete cut. Noting that the observed discharges were not ictal discharges but spontaneously repeating weaker epileptiform events, we have suggested that their synchronization occurs because of the potassium diffusion but with a delay longer than that of natural ID propagation. The propagation of activity between two parts of the slice may be mediated by the potassium diffusion, which is otherwise too slow to affect an ID. Our simulation with a complete lesion has confirmed this suggestion.

Analogy to spreading depression waves

Though we have found that potassium diffusion cannot be a major factor in the propagation of ictal wavefronts, the simulated slow waves in Model 1 are quite similar to concomitant waves of potassium and neuronal excitation observed in experimental models of spreading depression (SD) [43]. Obtained with a realistic potassium diffusion coefficient, the speed of waves is close to characteristic values of SD waves, of an order of a few mm/min [43, 44]. As is known [44], the mechanism of SD is also based on potassium diffusion, which explains the observed similarity. However, the details of our model should not be directly translated to the case of SD.

## Conclusion

The proposed model of ictal discharge generation and spread, found to be consistent with a majority of experimental observations, diminishes the role of potassium diffusion but supports the synaptic, potassium-mediated mechanism of ictal wavefront propagation. This prediction is important for the development of a treatment preventing seizure spread, and is to be further experimentally verified.

## References

[1] Trevelyan AJ, Sussillo D, Watson BO, Yuste R. Modular propagation of epileptiform activity: evidence for an inhibitory veto in neocortex. Journal of Neuroscience. 2006;26(48):12447–12455. 10.1523/JNEUROSCI.2787-06.2006 17135406 PMC6674895

[2] Trevelyan AJ, Sussillo D, Yuste R. Feedforward inhibition contributes to the control of epileptiform propagation speed. Journal of Neuroscience. 2007;27(13):3383–3387. 10.1523/JNEUROSCI.0145-07.2007 17392454 PMC6672122

[3] Smith EH, you Liou J, Davis TS, Merricks EM, Kellis SS, Weiss SA, et al The ictal wavefront is the spatiotemporal source of discharges during spontaneous human seizures. Nature Communications. 2016;7(1). 10.1038/ncomms11098 PMC4820627 27020798

[4] Schevon CA, Weiss SA, McKhann GJ, Goodman RR, Yuste R, Emerson RG, et al Evidence of an inhibitory constraint of seizure activity in humans. Nature Communications. 2012;3:1060 10.1038/ncomms2056 22968706 PMC3658011

[5] Schevon CA, Tobochnik S, Eiss T, Merricks E, Gill B, Parrish RR, et al Multiscale recordings reveal the dynamic spatial structure of human seizures. Neurobiology of Disease. 2019;127:303–311. 10.1016/j.nbd.2019.03.015 30898669 PMC6588430

[6] Wong BY, Prince DA. The lateral spread of ictal discharges in neocortical brain slices. Epilepsy Research. 1990;7(1):29–39. 10.1016/0920-1211(90)90051-v 1981355

[7] Weissinger F, Buchheim K, Siegmund H, Heinemann U, Meierkord H. Optical imaging reveals characteristic seizure onsets, spread patterns, and propagation velocities in hippocampal–entorhinal cortex slices of juvenile rats. Neurobiology of Disease. 2000;7:286–298. 10.1006/nbdi.2000.0298 10964601

[8] Weissinger F, Buchheim K, Siegmund H, Meierkord H. Seizure spread through the life cycle: Optical imaging in combinedbrain slices from immature, adult, and senile rats *in vitro*. Neurobiology of Disease. 2005;19:84–95. 10.1016/j.nbd.2004.11.013 15837564

[9] Cammarota M, Losi G, Chiavegato A, Zonta M, Carmignoto G. Fast spiking interneuron control of seizure propagation in a cortical slice model of focal epilepsy. Journal of Physiology. 2013;591(4):807–822. 10.1113/jphysiol.2012.238154 23207591 PMC3591699

[10] Wenzel M, Hamm JP, Peterka DS, Yuste R. Reliable and elastic propagation of cortical seizures *In Vivo*. Cell Reports. 2017;19(13):2681–2693. 10.1016/j.celrep.2017.05.090 28658617 PMC5551439

[11] Rossi LF, Wykes RC, Kullmann DM, Carandini M. Focal cortical seizures start as standing waves and propagate respecting homotopic connectivity. Nature Communications. 2017;8:217 10.1038/s41467-017-00159-6 28794407 PMC5550430

[12] Müller BJ, Zhdanov AV, Borisov SM, Foley T, Okkelman IA, Tsytsarev V, et al Nanoparticle-based fluoroionophore for analysis of potassium ion dynamics in 3D tissue models and *in vivo*. Advanced Functional Materials. 2018;28(9):1704598. 30271316 10.1002/adfm.201704598 PMC6157274

[13] Lian J, Bikson M, Shuai J, Durand DM. Propagation of non-synaptic epileptiform activity across a lesion in rat hippocampal slices. Journal of Physiology. 2001;537(1):191–199. 10.1111/j.1469-7793.2001.0191k.x 11711572 PMC2278941

[14] Vern BA, Schuette WH, Thibault LE. [K+]o clearance in cortex: a new analytical model. Journal of Neurophysiology. 1977;40(5):1015–1023. 10.1152/jn.1977.40.5.1015 143510

[15] Smith EH, you Liou J, Davis TS, Merricks EM, Kellis SS, Weiss SA, et al The extracellular space in the CNS: its regulation, volume and geometry in normal and pathological neuronal function. The Neuroscientist. 1997;3(1):28–41.

[16] Martinet LE, Fiddyment G, Madsen JR, Eskandar EN, Truccolo W, Eden UT, et al Human seizures couple across spatial scales through travelling wave dynamics. Nature Communications. 2017;8:14896 10.1038/ncomms14896 28374740 PMC5382286

[17] Wang Y, Trevelyan AJ, Valentin A, Alarcon G, Taylor PN, Kaiser M. Mechanisms underlying different onset patterns of focal seizures. PLOS Computational Biology. 2017;13(5):e1005475 10.1371/journal.pcbi.1005475 28472032 PMC5417416

[18] Chizhov AV, Amakhin DV, Zaitsev AV. Computational model of interictal discharges triggered by interneurons. PLOS ONE. 2017;12(10):e0185752 10.1371/journal.pone.0185752 28977038 PMC5627938

[19] Almeida AGD, Rodrigues AM, Scorza FA, Cavalheiro EA, Teixeira HZ, Duarte MA, et al Mechanistic hypotheses for nonsynaptic epileptiform activity induction and its transition from the interictal to ictal state—Computational simulation. Epilepsia. 2008;49:1908–1924. 10.1111/j.1528-1167.2008.01686.x 18513350

[20] Gentiletti D, Suffczynski P, Gnatkovsky V, de Curtis M. Changes of ionic concentrations during seizure transitions—a modeling study. Int J Neural Syst. 2017;27(4):1750004 10.1142/S0129065717500046 27802792

[21] Chizhov AV, Amakhin DV, Zaitsev AV. Mathematical model of Na-K-Cl homeostasis in ictal and interictal discharges. PLOS ONE. 2019;14(3):e0213904 10.1371/journal.pone.0213904 30875397 PMC6420042

[22] Wei Y, Ullah G, Schiff SJ. Unification of neuronal spikes, seizures, and spreading depression. Journal of Neuroscience. 2014;34(35):11733–11743. 10.1523/JNEUROSCI.0516-14.2014 25164668 PMC4145176

[23] Bazhenov M, Timofeev I, Steriade M, Sejnowski TJ. Potassium model for slow (2-3 Hz) *in vivo* neocortical paroxysmal oscillations. Journal of Neurophysiology. 2004;92(2):1116–1132. 10.1152/jn.00529.2003 15056684 PMC2925854

[24] Krishnan GP, Bazhenov M. Ionic dynamics mediate spontaneous termination of seizures and postictal depression state. Journal of Neuroscience. 2011;31(24):8870–8882. 10.1523/JNEUROSCI.6200-10.2011 21677171 PMC3163257

[25] Chizhov AV, Zefirov AV, Amakhin DV, Smirnova EY, Zaitsev AV. Minimal model of interictal and ictal discharges “Epileptor-2”. PLOS Computational Biology. 2018;14(5):e1006186 10.1371/journal.pcbi.1006186 29851959 PMC6005638

[26] Jirsa VK, Stacey WC, Quilichini PP, Ivanov AI, Bernard C. On the nature of seizure dynamics. Brain. 2014;137(8):2210–2230. 10.1093/brain/awu133 24919973 PMC4107736

[27] Proix T, Jirsa VK, Bartolomei F, Guye M, Truccolo W. Predicting the spatiotemporal diversity of seizure propagation and termination in human focal epilepsy. Nature Communications. 2018;9:1088 10.1038/s41467-018-02973-y 29540685 PMC5852068

[28] Olmi S, Petkoski S, Guye M, Bartolomei F, Jirsa V. Controlling seizure propagation in large-scale brain networks. PLoS Comput Biol. 2019;15(2):e1006805 10.1371/journal.pcbi.1006805 30802239 PMC6405161

[29] Jirsa VK, Haken H. Field theory of electromagnetic brain activity. Physical Review Letters. 1996;77(5):960–963. 10.1103/PhysRevLett.77.960 10062950

[30] Kager H, Wadman WJ, Somjen GG. Simulated seizures and spreading depression in a neuron model incorporating interstitial space and ion concentrations. Journal of Neurophysiology. 2000;84(1):495–512. 10.1152/jn.2000.84.1.495 10899222

[31] Cressman JR, Ullah G, Ziburkus J, Schiff SJ, Barreto E. The influence of sodium and potassium dynamics on excitability, seizures, and the stability of persistent states: I. Single neuron dynamics. Journal of Computational Neuroscience. 2009;26(2):159–170. 10.1007/s10827-008-0132-4 19169801 PMC2704057

[32] Cressman JR, Ullah G, Ziburkus J, Schiff SJ, Barreto E. Erratum to: The influence of sodium and potassium dynamics on excitability, seizures, and the stability of persistent states: I. Single neuron dynamics. Journal of Computational Neuroscience. 2011;30(3):781. 10.1007/s10827-008-0132-4 PMC2704057 19169801

[33] Loebel A, Tsodyks M. Computation by ensemble synchronization in recurrent networks with synaptic depression. J Comp Neuroscience. 2002;13:111–124. 10.1023/a:1020110223441 12215725

[34] Robinson PA, Rennie CJ, Wright JJ. Propagation and stability of waves of electrical activity in the cerebral cortex. Physical Review E. 1997;56(1):826–840.

[35] Izhikevich EM. Simple model of spiking neurons. IEEE Transactions on Neural Networks. 2003;14(6):1569–1572. 10.1109/TNN.2003.820440 18244602

[36] Raimondo JV, Burman RJ, Katz AA, Akerman CJ. Ion dynamics during seizures. Front Cell Neurosci. 2015;9:419 10.3389/fncel.2015.00419 26539081 PMC4612498

[37] Hodgkin AL, Keynes RD. The mobility and diffusion coefficient of potassium in giant axons from sepia. Journal of Physiology. 1953;119:513–528. 10.1113/jphysiol.1953.sp004863 13053453 PMC1392716

[38] Ma H, Zhao M, Schwartz TH. Dynamic neurovascular coupling and uncoupling during ictal onset, propagation, and termination revealed by simultaneous *in vivo* optical imaging of neural activity and local blood volume. Cerebral Cortex. 2012;23(4):885–899. 10.1093/cercor/bhs079 22499798 PMC3593576

[39] Kutsy RL, Farrell DF, Ojemann GA. Ictal patterns of neocortical seizures monitored with intracranial electrodes: correlation with surgical outcome. Epilepsia. 1999;40(3):257–266. 10.1111/j.1528-1157.1999.tb00702.x 10080503

[40] Blume WT, Ociepa D, Kander V. Frontal lobe seizure propagation: scalp and subdural EEG studies. Epilepsia. 2001;42(4):491–503. 10.1046/j.1528-1157.2001.26700.x 11440344

[41] Albowitz B, Kuhnt U. Predicting the spatiotemporal diversity of seizure propagation and termination in human focal epilepsy. Brain Research. 1993;631:329–333.8131062

[42] Trevelyan AJ, Baldeweg DT, van Drongelen W, Yuste R, Whittington M. The source of afterdischarge activity in neocortical tonic– clonic epilepsy. Journal of Neuroscience. 2007;27(49):13513–13519. 10.1523/JNEUROSCI.3005-07.2007 18057209 PMC6673106

[43] Whalen AJ, Xiao Y, Kadji H, Dahlem MA, Gluckman BJ, Schiff SJ. Control of spreading depression with electrical fields. Scientific Reports. 2018;8(1). 10.1038/s41598-018-26986-1 PMC5993812 29884896

[44] Ayata C, Lauritzen M. Spreading depression, spreading depolarizations, and the cerebral vasculature. Physiological Review. 2015;95:953–993. 10.1152/physrev.00027.2014 PMC4491545 26133935

